# siRNAEfficacyDB: An experimentally supported small interfering RNA efficacy database

**DOI:** 10.1049/syb2.12102

**Published:** 2024-11-14

**Authors:** Yang Zhang, Ting Yang, Yu Yang, Dongsheng Xu, Yucheng Hu, Shuo Zhang, Nanchao Luo, Lin Ning, Liping Ren

**Affiliations:** ^1^ Innovative Institute of Chinese Medicine and Pharmacy Academy for Interdiscipline Chengdu University of Traditional Chinese Medicine Chengdu China; ^2^ School of Healthcare Technology Chengdu Neusoft University Chengdu China; ^3^ School of Computer Science and Technology Aba Teachers College Aba Sichuan China

**Keywords:** big data, drugs, RNA

## Abstract

Small interfering RNA (siRNA) has revolutionised biomedical research and drug development through precise post‐transcriptional gene silencing technology. Despite its immense potential, siRNA therapy still faces technical challenges, such as delivery efficiency, targeting specificity, and molecular stability. To address these challenges and facilitate siRNA drug development, we have developed siRNAEfficacyDB, a comprehensive database that integrates experimentally validated siRNA efficacy data. This database contains 3544 siRNA records, covering 42 target genes and 5 cell lines. It provides detailed information on siRNA sequences, target genes, inhibition efficiencies, experimental techniques, cell lines, siRNA concentrations, and incubation times. siRNAEfficacyDB offers a user‐friendly web interface that makes it easy to query, browse and analyse data, enabling efficient access to siRNA‐related information. In summary, siRNAEfficacyDB provides a useful data foundation for siRNA drug design and optimisation, serving as a valuable resource for advancing computer‐aided siRNA design research and nucleic acid drug development. siRNAEfficacyDB is freely available at https://cellknowledge.com.cn/siRNAEfficacy for non‐commercial use.

## Graphical abstract

1. siRNAEfficacyDB integrates experimentally validated siRNA efficacy data, providing detailed information on 3544 siRNA records. 2. The database features a user‐friendly web interface for easy querying, browsing, and analysis of siRNA‐related information. 3. siRNAEfficacyDB serves as a valuable resource for advancing computer‐aided siRNA design research and nucleic acid drug development.

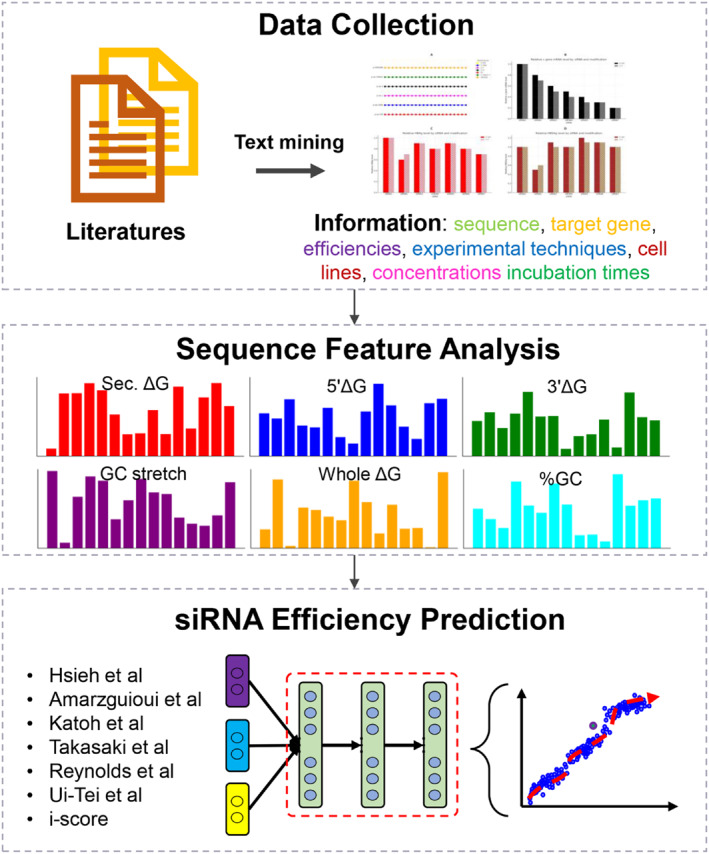

## 1 INTRODUCTION

**FIGURE 1:**
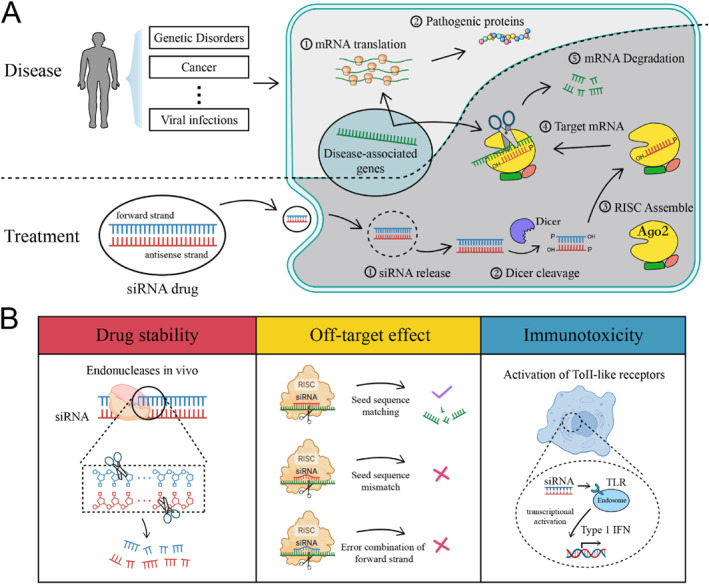
Principles and Challenges of Small interfering RNA (siRNA) Molecular Therapy. (a) Molecular Mechanism of siRNA Therapy. (b) Three Challenges in siRNA Drug Development.

Small interfering RNA (siRNA) has emerged as a crucial post‐transcriptional gene silencing tool in biomedical research and drug development [1]. Since Andrew Fire and Craig Mello elucidated the mechanism of double‐stranded RNA‐mediated gene silencing in 1998 [2], siRNA technology has been widely recognised as a revolutionary therapeutic approach with significant potential [3]. Compared to traditional drug therapies, siRNA offers several advantages, including shorter development cycles, lower costs, broad target range, and well‐defined mechanisms, enabling targeted regulation of protein targets that are insensitive to conventional small molecule drugs (Figure 1a) [1, 3]. However, despite its theoretical promise, the clinical efficacy of siRNA remains constrained by numerous biological and technical challenges, including drug delivery efficiency, targeting specificity, molecular stability, and potential off‐target effects (Figure 1b) [4, 5, 6, 7].

Ensuring the efficient and precise silencing of target genes is critical in siRNA drug development, as it not only determines therapeutic efficacy but also impacts drug safety. The design and optimisation of siRNA molecules involve a complex process with numerous variables [8, 9, 10]. From selecting the appropriate target sequence to designing suitable delivery systems, each step requires meticulous control of biochemical parameters. For instance, the clinical application of first‐generation siRNA drugs is limited by inadequate delivery efficiency and potential immune activation issues [11]. Additionally, to minimise off‐target effects, ideal siRNA design must ensure high specificity for the target gene, thereby avoiding unintended interference with non‐target genes [12]. These technical challenges increase the complexity and cost of the development process, necessitating continuous innovation and technological improvements [13].

In the process of siRNA design and optimisation, studies have demonstrated that targeting different sequences within the same mRNA can lead to significant variations in knockdown efficiency [14, 15]. For instance, Khvorova et al. [16] highlighted that functional siRNAs exhibit a pronounced strand bias, meaning that the antisense strand preferentially guides the RNA‐induced silencing complex, and this bias is closely linked to the overall efficacy of the siRNA. Similarly, Reynolds et al. [17] proposed a set of rational siRNA design rules, underlining the importance of specific sequence features in predicting siRNA activity. Additionally, Ui‐Tei et al. [18] provided comprehensive guidelines for selecting highly effective siRNA sequences in mammalian and avian systems, further refining the standards for siRNA design. In the same year, Amarzguioui and Prydz [19] introduced an algorithm for functional siRNA selection that combined sequence characteristics with positional effects, significantly improving the success rate of siRNA design. This collective body of work has established a strong foundation for understanding how sequence and positional factors critically influence siRNA efficacy, laying the groundwork for more sophisticated design approaches in RNA interference research.

With the explosive growth of machine learning technologies, particularly deep learning‐based artificial intelligence, using machine learning methods to predict siRNA activity and assist in siRNA drug design has become an emerging strategy in nucleic acid drug development [20, 21, 22]. Compared to traditional research methods, machine learning offers advantages, such as accurate prediction and optimisation, reduced experimental costs, automation, increased efficiency, and personalised customisation [22]. In the era of traditional machine learning, Huesken et al. [23] first established a dataset containing 2431 siRNA activity records and developed the BIOPREDsi algorithm using the Stuttgart neural network simulator. Ichihara et al. [24] developed a simple linear regression model called i‐Score, focussing on predicting active siRNAs by considering nucleotide preferences at each position within the siRNA sequence. Lu et al. [25, 26] constructed a support vector machine model combining thermodynamic and sequence features specifically for selecting functional siRNAs.

In recent years, researchers have started applying deep learning models to predict siRNA activity. For example, Han et al. [27] used convolution kernels as motif detectors to extract features from siRNA sequences, combined with thermodynamic properties and pooling layers, and introduced deep neural networks to predict siRNA activity. Massimo et al. [28] proposed a graph neural network method to model siRNA–mRNA interactions and predict siRNA activity. Additionally, Bai et al. [29] further optimised siRNA efficiency prediction by integrating thermodynamic calculations, RNA‐FM modules(the first foundation model for the community to accommodate all non‐coding RNA sequences), and Oligo encoders representing siRNA.

In the field of siRNA research, several databases have provided researchers with valuable resources on siRNA efficacy, supporting the design and optimisation of siRNA. For instance, siRNAdb has compiled extensive experimental validation data on functional siRNAs, including detailed information on sequences, target genes, and experimental conditions, making it a critical reference for siRNA function and design [30]. Similarly, siRecords offers a broad collection of siRNA experimental records, covering multiple biological systems and documenting gene knockdown efficiencies, along with multi‐level evaluations of siRNA efficacy, which assist researchers in screening and analysing siRNA biological activity [31]. VIRsiRNAdb, on the other hand, focuses on virus‐related siRNA data, containing siRNA sequences targeting viral genomes and their inhibitory efficiencies, advancing the development of antiviral siRNA therapies [32]. However, these databases are no longer accessible online, forcing researchers to rely on diverse and dispersed sources for training data, lacking unified management and curation [33]. This significantly increased the difficulty of training machine learning algorithms and hindered their generalisation capabilities. To address this issue, our study leveraged literature mining techniques to construct siRNAEfficacyDB, a database containing 3544 experimentally validated siRNA efficiency records. We also developed a user‐friendly online platform, enabling researchers and developers to easily access and study detailed information on siRNA efficacy. The establishment of this database not only provides robust data support for siRNA drug design and optimisation but also holds significant scientific and practical value in advancing nucleic acid drug development.

## 2 MATERIALS AND METHODS

### 2.1 Data collection

**FIGURE 2:**
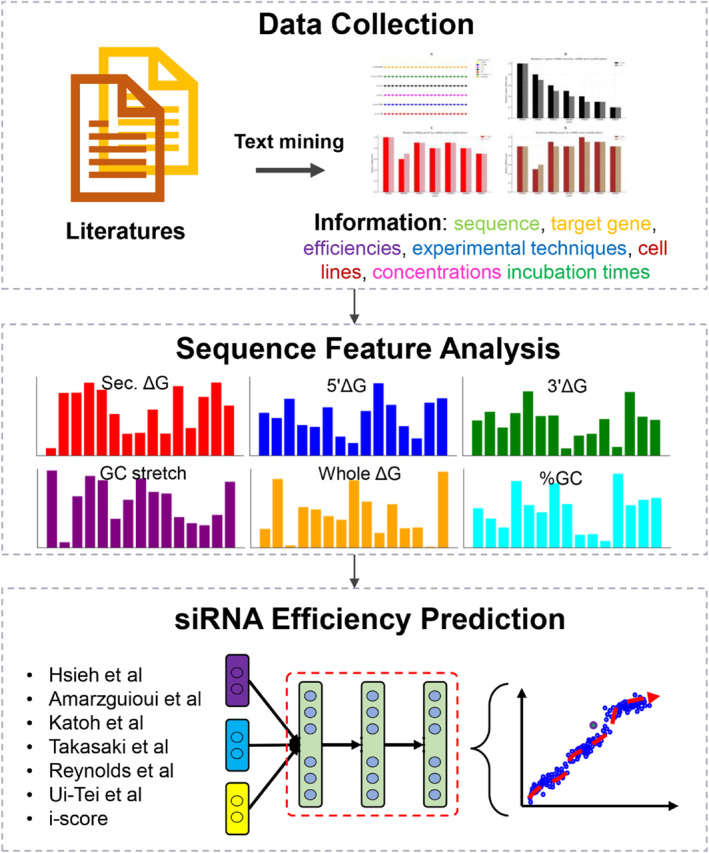
Small interfering RNA (siRNA) data collection, Sequence Feature Analysis and Efficiency Prediction in siRNAEfficacyDB.

All data in siRNAEfficacyDB were sourced from seven publications (Figure 2). Specifically, 2431 entries were obtained from Huesken et al. [23], 44 entries from Harborth et al. [34], 10 entries from Khovorova et al. [16], 244 entries from Reynolds et al. [17], 702 entries from Takayuki Katoh et al. [35], 37 entries from Ui‐Tei et al. [18], and 76 entries from Vickers et al. [36]. We collected information on the sense and antisense sequences of all siRNAs, target genes, inhibition efficiencies, experimental techniques, cell lines, siRNA concentrations, and incubation times.

To ensure data reliability, only siRNA records meeting the following criteria were included in the database: Experimental Validation: All siRNAs must have been experimentally tested, with reported measurable inhibition efficiencies. Data Completeness: Each record must include both sense and antisense siRNA sequences, along with associated experimental conditions (e.g. cell type, siRNA concentration, and incubation times). Relevance to siRNA Design: Only studies contributing to the understanding of siRNA design, mechanism of action, or gene silencing optimisation were considered. By adhering to these criteria, siRNAEfficacyDB offers a high‐quality, standardised dataset that supports siRNA drug design and optimisation, providing a solid foundation for training models that predict siRNA efficacy.

### 2.2 Small interfering RNA sequence feature analysis

In this study, we further analysed six fundamental features of all siRNA sequences using the i‐Score [24] platform (Figure 2):

Sec. dG: The Δ*G* value of the most stable secondary structure of the siRNA strand;
5′Δ*G*: The Δ*G* value of the dinucleotide at the 5′ end;
3′Δ*G*: The Δ*G* value of the dinucleotide at the 3′ end;
Whole Δ*G*: The Δ*G* value of the double‐stranded siRNA;
GC stretch: The maximum length of the GC segment;
%GC: The percentage of GC content.

### 2.3 Prediction of small interfering RNA inhibition efficiency

In addition to providing experimentally obtained siRNA inhibition efficiencies, we also predicted the efficiencies of all siRNAs in the database using seven siRNA efficiency prediction algorithms (Figure 2): Hsieh et al. [23], Amarzguioui et al. [19], Katoh et al. [35], Takasaki et al. [37], Reynolds et al. [17], Ui‐Tei et al. [18], and i‐score [24].

### 2.4 Website architecture

**FIGURE 3:**
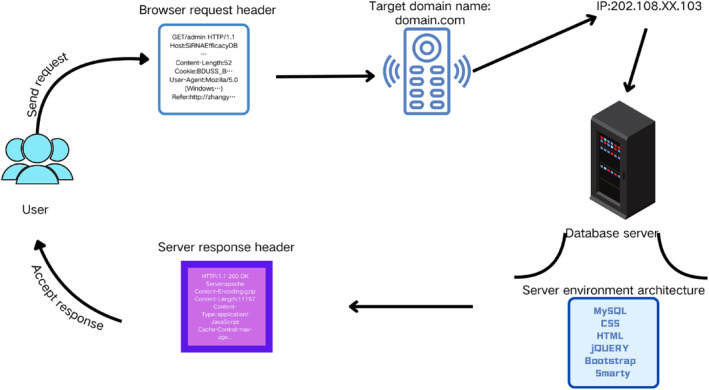
Architecture of siRNAEfficacyDB.

The system adopts a three‐tier architecture comprising the presentation layer, logic layer, and data layer (Figure 3). The presentation layer, developed using HTML, CSS, and JavaScript, offers a user‐friendly interface and interactive experience. The logic layer, implemented in PHP, handles user requests, invokes data, and implements system functionalities, with AJAX technology used for real‐time data retrieval. The data layer utilises MySQL for data management and storage. The Smarty template engine separates the front end from the back end, facilitating maintenance and updates. The website features a responsive design, adapting to different screen sizes and devices to enhance the user browsing experience.

## 3 RESULTS

### 3.1 Statistics

**FIGURE 4:**
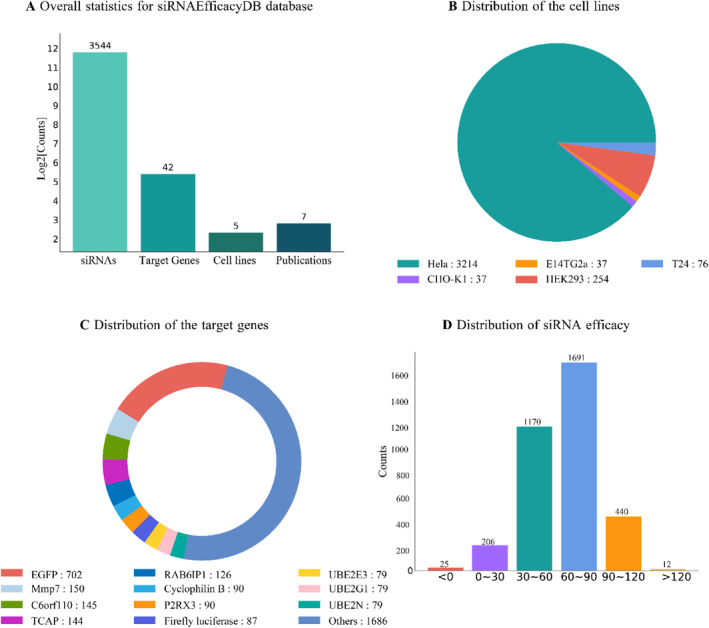
Data statistics of siRNAEfficacyDB. (a) Overall statistics for siRNAEfficacyDB database. (b) Distribution of cell lines. (c) Distribution of target genes. (d) Distribution of Small interfering RNA (siRNA) efficacy.

The current version of siRNAEfficacyDB includes 3544 siRNA entries sourced from seven publications, involving 42 target genes and 5 cell lines (Figure 4a). Over 90% of the data are derived from experiments conducted on the HeLa cell line (Figure 4b). Among the 42 target genes, a significant proportion are various fluorescent proteins and enzyme genes, such as Enhanced Green Fluorescent Protein, Ubiquitin Conjugating Enzyme E2 E3, and matrix metallopeptidase 7 (Figure 4c). Further analysis of the inhibition efficiencies of all siRNAs reveals that their efficiencies predominantly range between 30% and 90% (Figure 4d).

### 3.2 Data querying and result presentation

**FIGURE 5:**
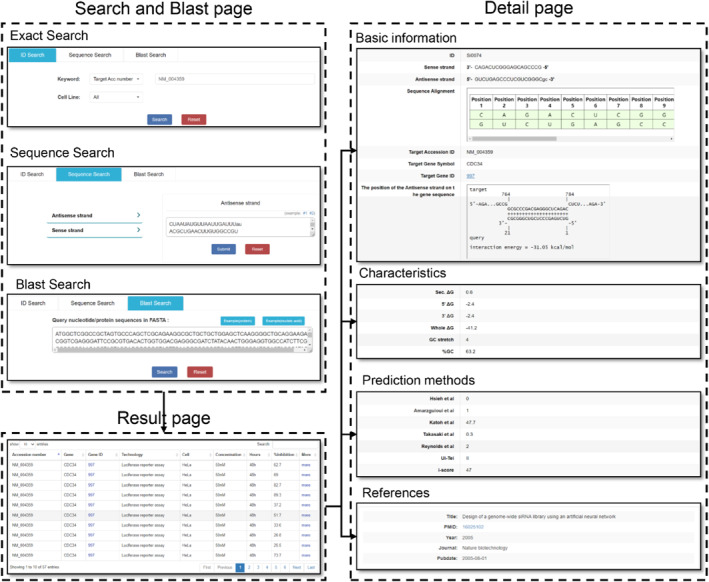
The Search page, Result page and Detail page of siRNAEfficacyDB.

siRNAEfficacyDB provides a user‐friendly web interface that allows users to easily query information related to siRNAs and their inhibition efficiencies. The website's navigation bar offers quick access to various pages, including “Search,” “Browse,” “Submit,” “Download,” and “Statistics.” The “Search” page offers three search options: “ID Search,” where users can enter gene ID/Accession/symbol; “Sequence Search,” where users can input siRNA sequences in bulk; and “Basic Local Alignment Search Tool Search,” where users can input specific sequences to retrieve similar target gene information. Search results are summarised in a table on the “Results” page (Figure 5). By clicking “More,” users can access detailed information about specific siRNA entries on the “Details” page. The “Details” page provides comprehensive information related to the siRNA, including basic information (such as “Antisense Strand,” “Sense Strand,” “Sequence Alignment,” “Target Gene,” “Technology,” “Cell Line,” “Concentration,” " Incubation Time,” and " Inhibition Efficacy”), characteristics (including “5′ end Δ*G*," ”3′ end Δ*G*," “Sec. Δ*G*,” “Whole Δ*G*,” “GC Stretch,” and "%GC”), prediction methods (including “Hsieh et al.” “Amarzguioui et al.” “Katoh et al.” “Takasaki et al.” “Reynolds et al.” “Ui‐Tei,” and “i‐score”), and references associated with the entry (including “PMIDs,” “Title,” “Journal,” and publication year). Overall, siRNAEfficacyDB offers a comprehensive and user‐friendly platform for users to efficiently access information related to siRNA efficiency.

### 3.3 Other interfaces

**FIGURE 6:**
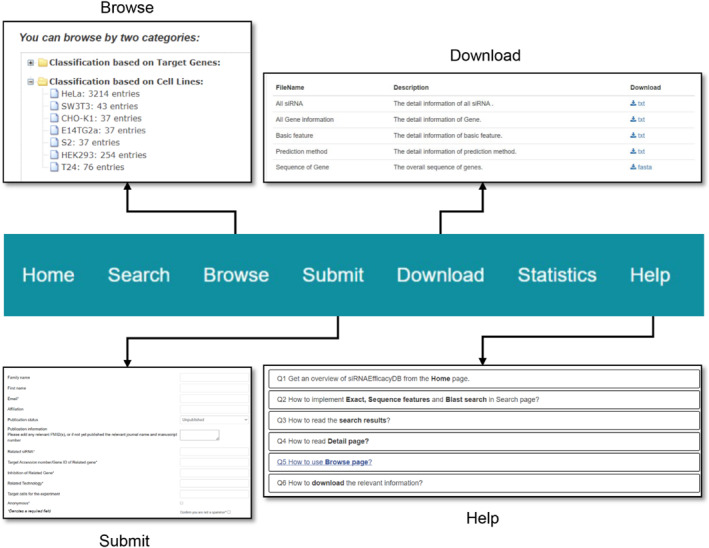
The Browse page, download page, submit and help page of siRNAEfficacyDB.

siRNAEfficacyDB features a “Browse” page that helps users quickly navigate siRNA data by selecting specific target genes, cell lines and experimental techniques (Figure 6). Users can then click on each entry in the “Results” table to view detailed information. The “Download” page allows users to easily download siRNA, target gene information, siRNA sequence features, and efficiency prediction results. The “Statistics” page uses various statistical charts to display detailed information about the latest version of siRNAEfficacyDB. The “Help” page provides step‐by‐step tutorials guiding users on how to operate, query, and browse the siRNAEfficacyDB database. Since siRNAEfficacyDB may not cover all reported siRNA efficacy data, a “Submit” interface is provided, allowing researchers to submit new siRNA efficacy information not recorded in the database. When users submit new siRNA efficacy data, they are required to provide detailed information about the data's source (e.g. specific references to published literature or patents). Our research team will periodically review all submissions to verify the reliability of the data sources and the accuracy of the submitted information. This review process includes cross‐checking the data against the provided references and ensuring that the methodology aligns with established standards in siRNA research. Only after this thorough validation process will the submitted data be added to the database in the next scheduled update, ensuring that the data remain of high quality and useable for the broader scientific community.

## 4 DISCUSSION

siRNAEfficacyDB provides a valuable platform for storing, integrating, and analysing siRNA efficacy and related information to accelerate the siRNA drug development process. However, it still has some limitations. Currently, the collected siRNA efficacy data cover only 42 target genes, which is very limited compared to the vast genome. This limitation means that when training related artificial intelligence models, we will still face the problem of insufficient generalisation ability. Secondly, all siRNA inhibition efficiency data are derived from cell experiments, lacking in vivo experimental results. Therefore, the data cannot be used to predict siRNA efficiency in living organisms. Additionally, chemical modifications on siRNA sequences significantly impact their efficiency, and the current database lacks data collection and organisation related to various chemically modified siRNAs.

To enhance the scientific value of siRNAEfficacyDB, we plan the following improvements: Expand target gene coverage: By collecting more siRNA efficacy data across a wider range of target genes, we aim to improve the generalisability of predictive models and cover a broader portion of the genome. Incorporate in vivo data: Adding in vivo experimental data will enhance the translational relevance of the database, helping predict siRNA performance in clinical settings. Integrate with other databases: Merging with other siRNA‐related databases will create a more comprehensive resource, offering researchers better support for designing and optimising siRNA therapeutics. Support chemically modified siRNAs: Including data on chemically modified siRNAs will help researchers understand the effects of modifications on efficacy, stability, and safety, improving drug development. Develop predictive tools: Using the expanded dataset, we will create deep learning‐based models to predict siRNA efficacy, boosting efficiency in drug development and personalised medicine. We believe that these efforts will position siRNAEfficacyDB as a key resource for advancing both basic research and therapeutic development in the siRNA field.

## 5 CONCLUSION

In this study, we collected 3544 experimentally validated siRNA activity data entries by mining and organising a large number of literature sources and successfully established the siRNAEfficacyDB. These data encompass key information such as siRNA sequence characteristics, target gene information, experimental conditions, and activity indicators. This provides a rich data foundation for siRNA drug design and optimisation, creating a valuable resource‐sharing bridge for small nucleic acid drug development and advancing the research of computer‐aided siRNA design.

## AUTHOR CONTRIBUTIONS

YZ, HL, LN and LPR conceived and designed the experiments. YZ, TY, YY and DSX performed the experiments. YCH, SZ and PLC analysed the data. YZ, HL, LPR wrote the paper. All authors discussed the results and commented on the manuscript.

## CONFLICT OF INTEREST STATEMENT

The authors declare that the research was conducted in the absence of any commercial or financial relationships that could be construed as a potential conflict of interest.

## Data Availability

All data in siRNAEfficacyDB complies with the Creative Commons Attribution Non‐Commercial License (http://creativecommons.org/licenses/by‐nc/2.0/uk/), which permits unrestricted non‐commercial use, distribution, and reproduction in any medium, provided the original work is properly cited.
